# [Expression of Concern] Ginsenoside Rg1 protects against H_2_O_2_-induced neuronal damage due to inhibition of the NLRP1 inflammasome signalling pathway in hippocampal neurons *in vitro*

**DOI:** 10.3892/ijmm.2026.5988

**Published:** 2026-09-16

**Authors:** Tan-Zhen Xu, Xiao-Yan Shen, Ling-Ling Sun, Ya-Li Chen, Bi-Qiong Zhang, Da-Ke Huang, Wei-Zu Li

Int J Mol Med 43: 717-726, 2019; DOI: 10.3892/ijmm.2018.4005

Following the publication of the above paper, it was drawn to the Editor's attention by an interested reader that an unsuitable antibody may have been used as a part of the western blot analysis shown in Fig. 3A on p. 720. The anti-β-galactosidase antibody (cat. no. ab9361; Abcam) that was employed, which is specific for the *E. coli* β-galactosidase encoded by the *LacZ* gene; is only distantly related to the human protein, and would not necessarily be expected to bind to it.

The authors have been contacted by the Editorial Office to offer an explanation for the selection of this apparently unsuitable antibody for the western blot analysis, and we are awaiting their response. Owing to the fact that the Editorial Office has been made aware of a potential problem surrounding the design of this experiment in this study, we are issuing an Expression of Concern to notify readers of this issue while the Editorial Office continues to investigate this matter further.

